# Possible Effects and Mechanisms of Dietary Natural Products and Nutrients on Depression and Anxiety: A Narrative Review

**DOI:** 10.3390/antiox11112132

**Published:** 2022-10-28

**Authors:** Si-Xia Wu, Jiahui Li, Dan-Dan Zhou, Ruo-Gu Xiong, Si-Yu Huang, Adila Saimaiti, Ao Shang, Hua-Bin Li

**Affiliations:** 1 Guangdong Provincial Key Laboratory of Food, Nutrition and Health, Department of Nutrition, School of Public Health, Sun Yat-Sen University, Guangzhou 510080, China; 2 School of Science, The Hong Kong University of Science and Technology, Hong Kong 999077, China; 3 School of Chinese Medicine, Li Ka Shing Faculty of Medicine, The University of Hong Kong, Hong Kong 999077, China

**Keywords:** dietary natural products, nutrients, depression, anxiety, gut microbiota

## Abstract

Depression and anxiety are severe public health problems and have attracted more and more attention from researchers of food science and nutrition. Dietary natural products and nutrients, such as fish, coffee, tea, n-3 PUFA, lycopene, and dietary fiber, could play a vital role in the prevention and management of these diseases. The potential mechanisms of action mainly include inhibiting inflammation, ameliorating oxidative stress, modulating the microbiota–gut–brain axis, suppressing hypothalamic–pituitary–adrenal axis hyperactivity, and regulating the levels of monoamine neurotransmitters. In this narrative review, we summarize the most recent advancements regarding the effects of dietary natural products and nutrients on depression and anxiety, and their underlying mechanisms are discussed. We hope that this paper can provide a better understanding of the anti-depressive and anxiolytic action of dietary natural products, and that it is also helpful for developing dietary natural products for functional food, dietary supplements, or auxiliary agents for the prevention and management of these diseases.

## 1. Introduction

Neuropsychiatric disease is a serious public health problem in the world. Depression and anxiety are two of the most common neuropsychiatric diseases, and they usually occur simultaneously or sequentially [1]. There are nearly 322 million people suffering from depression in the world and it has become the second leading cause of years lost to disability worldwide [2,3]. Depression is characterized by fatigue, anhedonia, sleep disturbance, and self-destructive behavior, which seriously affect patients’ mental health and social functions [4]. In addition, anxiety affects more than 260 million people worldwide according to a report from the World Health Organization [3]. Individuals with anxiety may experience dizziness, headaches, shortness of breath, fatigue, nausea, palpitations, or urinary incontinence, which could lead to severe psychological distress, significant disability, and a reduction in their quality of life [5].

The pathogenesis of depression and anxiety is very complicated. Accumulating evidence has suggested that the occurrence of depression is associated with many factors, such as weakened levels of neurotransmitters, the hyperactivity of the hypothalamic–pituitary–adrenal (HPA) axis, disorders of gut microbiota, oxidative stress, and neuroinflammation [6,7]. Gut microbiota disorders and gut–brain axis dysfunction in particular play an important role in the development of depression [8,9]. The alteration of gut microbiota diversity could change gut barrier permeability, stimulate inflammation and immune responses, affect the release and efficacy of monoamine neurotransmitters, and decrease the expression level of brain-derived neurotrophic factor (BDNF), which may provoke depressive episodes [10]. Additionally, anxiety is correlated with inflammation and oxidative stress as well as the neuronal noradrenergic and serotonergic dysfunction of the central nervous system [11,12]. The excessive secretion of adrenal hormones and pro-inflammatory cytokines could inhibit the expression of BDNF in the brain, which may accelerate the occurrence of anxiety [13].

Psychotherapy and drug treatment are the main therapeutic measures for depression and anxiety, but their effectiveness is limited by low tolerance and some adverse effects [12]. Therefore, it is important to search for novel strategies for the prevention and treatment of these diseases. Several studies have reported that exercise and sunlight exposure could improve depressive symptoms and decrease the risk of depression [14,15,16]. Furthermore, the anti-depressive and anxiolytic effects of dietary natural products and nutrients have currently become a research hotspot. An increasing number of studies have shown that dietary natural products and nutrients, such as tea, vegetables, fruits, medicinal plants, and curcumin, could inhibit inflammation and oxidative stress and improve the function of the nervous system as well as modulate biomarkers and signaling pathways associated with depression and anxiety [7,17,18]. For example, purple cauliflower improved depressive symptoms through upregulating the expression levels of related biomarkers, such as BDNF and monoamine neurotransmitters [19]. It was found that honey had protective effects against anxiety through the suppression of inflammation responses by decreasing the expression level of pro-inflammatory cytokines [20]. Thus, we searched for relevant high-quality papers from the Core Collection of Web of Science and PubMed from the last five years based on keywords in the titles and abstracts, including depression, anxiety, vegetable, fruit, spice, tea, grain, cereal, medicinal herb, probiotics, prebiotics, meat, fish, and food, in order to better understand the anti-depressive and anxiolytic effects of dietary natural products and nutrients. In this narrative review, we summarize the effects of dietary natural products and nutrients on depression and anxiety based on the results from epidemiological, experimental, and clinical studies. In addition, special attention is paid to the underlying mechanisms of action.

## 2. Depression

The pathology of depression was associated with neuroinflammation, monoamine deficiency, gut microbiota disorder, the hyperactivity of the HPA axis, and so on [10,21]. Many studies have shown that some dietary natural products and nutrients could exert anti-depressive effects through different mechanisms [22,23].

### 2.1. Epidemiological Studies

A number of epidemiological studies have shown that several dietary natural products and nutrients are associated with the risk of depression (Table 1). For example, a prospective cohort study of 3177 Asian older adults showed that those who drank ≥3 cups of tea daily had a lower risk of depression (odds ratio (OR), 0.32; 95% confidence interval (CI), 0.12–0.84), compared with those who did not drink tea [24]. Another study of 9576 Korean adults found ≥3 cups/week of green tea decreased the risk of self-reported lifetime depression [25]. A cross-sectional survey found that a low intake of dietary fiber was associated with a high incidence of depression in hypertensive patients. Specifically, compared with those who consumed dietary fiber ≥ 15.4 g/day, the OR of 10.5–15.4 g/day to incidence of depression was 2.641 (95% CI, 1.050–6.640) [26]. In addition, a cross-sectional study of 736 Brazilian farmers showed that the higher consumption of selenium decreased the risk of depression by 54% after adjusting for sociodemographic variables, pesticide intoxication, and lifestyle [27]. A meta-analysis of 16 cross-sectional and 9 cohort studies showed that each 100 g increase of fruit intake decreased the risk of depression by 3% in cohort studies (risk ratio (RR), 0.97; 95% CI, 0.95–0.99), and each 100 g increase of vegetable consumption reduced the risk of depression by 5% in cross-sectional studies (RR, 0.95; 95% CI, 0.91–0.98) [28]. Additionally, a higher intake of seaweed (OR, 0.38; 95% CI, 0.20–0.72) and mushroom fiber (OR, 0.18; 95% CI, 0.01–0.37) were negatively associated with depressive symptoms [29]. Daily walnut consumption decreased the risk of depression with an OR of 0.67 (95% CI, 0.48–0.93), and this effect was found mainly in women (OR, 0.62; 95% CI, 0.46–0.84) but not in men (OR, 0.72; 95% CI, 0.41–1.27) [30]. The exact reason for the gender difference remains unknown, and might have been a dose effect in which women consumed more walnuts. A cross-sectional study of 9183 Korean adults revealed that the prevalence of depression was decreased by 48% when people consumed ≥4 times of fish weekly, and the protective effect was particularly found in women (OR, 0.44; 95% CI, 0.29–0.67) but not in men (OR, 0.64; 95% CI, 0.30–1.37) [31]. The reason for this gender difference might be biological differences between men and women, such as differences in sex hormones. Furthermore, a study showed there was a U-shaped relationship between fatty fish consumption and the risk of depression. In other words, moderate fatty fish consumption, but not high consumption, was associated with a lower risk of depression [32]. Perhaps the high consumption of fatty fish may lead to obesity, which is associated with the occurrence of depression. Moreover, those who drank ≥4 cups of coffee daily had a lower risk of depression, compared with those who drank <1 cup/day (hazard ratios (HR), 0.37; 95% CI, 0.15–0.95) [33]. A two-sample bidirectional Mendelian randomization analysis showed that there was a causal relationship between the higher consumption of carbohydrates and a lower risk of depression (OR, 0.42; 95% CI, 0.28–0.62) [34].

It should also be pointed out that inconsistent results have been reported and not all studies showed protective effects of dietary natural products against depression. For example, a cross-sectional study found no link between legume and nut consumption and depression either in men (OR, 0.96; 95% CI, 0.54–1.71) or women (OR, 0.98; 95% CI, 0.65–1.48) [35]. Soft drink is a kind of drink containing a great number of added sugars and mainly includes syrup, soda water, and other carbonated or non-carbonated beverages. A cross-sectional study of 8085 Chinese college students found that those who consumed >25 g sugar/day from soft drinks had an increased risk of depression compared with those who did not drink soft drinks [36]. However, another study of 15,546 Spanish university graduates showed that there was no association between sugar-sweetened beverage consumption and the risk of depression (HR, 1.12; 95% CI, 0.90–1.41) [37]. The inconsistent results from the different studies might be due to a racial factor (Chinese vs. Spanish).

In brief, many studies have pointed out that some dietary natural products could decrease the risk of depression, such as fish, walnuts, tea, and coffee. However, several studies have found no link between some dietary natural products and depression, such as legumes and nuts. In addition, inconsistent results regarding soft drink consumption and depression were reported by different studies, which should be further studied in the future. These results from epidemiological studies only indicated the possible anti-depressive effects of these substances, and clinical trials should be carried out to verify their effects on human beings. Furthermore, cell and animal experiments also should be performed to investigate the possible mechanisms of these substances on depression.

### 2.2. Experimental Studies

**Figure 1:**
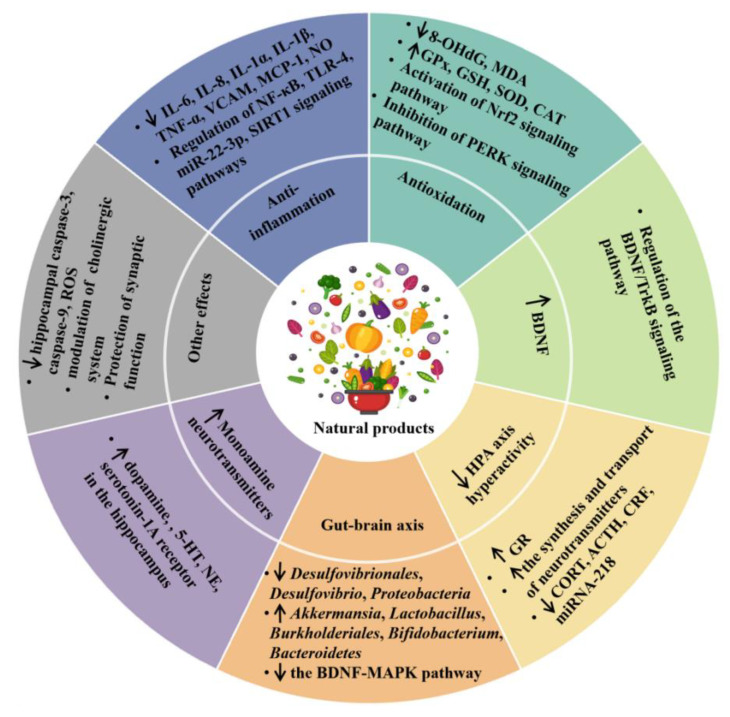
The effects and mechanisms of dietary natural products on depression. ↑ represents increase, and ↓ represents decrease. ACTH, adrenocorticotropic hormone; BDNF, brain-derived neurotrophic factor; CAT, catalase; CORT, corticosterone; CRF, corticotropin-releasing factor; GPx, glutathione peroxidase; GR, glucocorticoid receptor; GSH, glutathione; 5-HT, serotonin; IL, interleukin; MAPK, mitogen-activated protein kinase; MCP-1, macrophage chemoattractant protein 1; MDA, malondialdehyde; NE, norepinephrine; NF-κB, nuclear factor kappa-B; NO, nitric oxide; Nrf2, nuclear erythroid related factor 2; 8-OHdG, 8-hydroxydeoxyguanosine; PERK, protein kinase-like endoplasmic reticulum kinase; ROS, reactive oxygen species; SCFA, short-chain fatty acids; SIRT1, Sirtuin 1; TNF-α, tumor necrosis factor-α; SOD, superoxide dismutase; TrkB, tyrosine kinase receptor B; VCAM, vascular cell adhesion molecule.

Many studies showed that some dietary natural products and nutrients had protective effects against depression through different mechanisms, including an anti-inflammatory effect, antioxidant effect, promoting the production of monoamine neurotransmitters, normalizing the hyperactivity of the HPA axis, regulating the microbiota–gut–brain axis, and other mechanisms [7,38,39], which are discussed in detail below (Table 2 and Figure 1).

#### 2.2.1. Anti-Inflammatory Effect

Neuroinflammation is one of the most important risk factors for depression [40]. Microglial cell activation could lead to neuroinflammation and increase the production of inflammatory cytokines, which results in neuronal damage and the progress of depression [41]. It was found that n-3 polyunsaturated fatty acids (PUFA) could downregulate the expression of interleukin (IL)-6, IL-1β, tumor necrosis factor (TNF)-α, and prostaglandin E-2, which ameliorated post-menopausal depression induced by chronic mild stress and maternal separation [42]. In addition, *Saccharina japonica* is a common marine vegetable in East Asia and its ethanol extract decreased the depressive symptoms of dextran sodium sulfate-induced mice by increasing anti-inflammatory cytokine and downregulating expression levels of nuclear factor kappa-B (NF-κB), NOD-like receptor 3, and Toll-like receptor-4 (TLR-4) [43]. Furthermore, apple phenolic extract improved lead acetate-induced depression-like behaviors through attenuating neuroinflammation and neuronal apoptosis, which was associated with the miR-22-3p/sirtuin 1 (SIRT1) signaling pathway [39]. In an ovariectomy mice model, the oral application of *Prevotella histicola* improved estrogen deficiency-induced depression through downregulating the expression levels of the vascular cell adhesion molecule (VCAM), macrophage chemoattractant protein 1 (MCP-1), IL-6, IL-8, and TNF-α in the ileum and colon of mice [44]. Moreover, *Lacticaseibacillus paracasei* NK112 exerted protective effects against *Escherichia coli*-induced depression through decreasing the expression of IL-1α, IL-6, and TNF-α, and inhibiting the activity of NF-κB in the hippocampus [45]. In a rat model of chronic unpredictable mild stress (CUMS)-induced depression, epigallocatechin gallate (EGCG) exerted an anti-depressive effect by reducing IL-6 and nitric oxide (NO) expression levels and decreasing the mRNA expression of caspase-3 and caspase-9 in the hippocampus [46].

In a word, *Saccharina japonica*, apple phenolic extract, *Prevotella histicola, Lacticaseibacillus paracasei* NK112, n-3 PUFA, and EGCG could decrease depression through anti-inflammatory action in preclinical models, which should be verified in clinical trials.

#### 2.2.2. Antioxidant Effect

Oxidative stress could disrupt the balance between oxidation and antioxidative defense and impair the structure and function of neural cells, which was closely associated with the development of depression [47]. A number of studies have shown that some dietary natural products and nutrients have antioxidant activity that attenuates depression-like behaviors. For example, lycopene improved oxidative stress and endoplasmic reticulum stress-induced damage on neuroblastoma cells by reducing expression levels of 8-hydroxydeoxyguanosine, malondialdehyde (MDA), and protein carbonyls and inhibiting the protein kinase-like endoplasmic reticulum kinase (PERK) signaling pathway [48]. *Grewia asiatica* berry is a popular berry in Pakistan that is mainly consumed in the form of carbonated drinks and fresh juices and is rich in phenols, anthocyanins, vitamin C, and flavonoids [49]. *Grewia asiatica* berry juice could improve depressive symptoms by decreasing oxidative damage in the brain through increasing superoxide dismutase (SOD) and glutathione peroxidase (GPx) levels [50]. Another study pointed out that *Saccharina japonica* ethanol extract decreased the depression-like behaviors of dextran sodium sulfate-induced mice by increasing the activity of SOD [43]. In addition, maqui berries had an anti-depressive effect against post-stroke depression by upregulating expression levels of reduced glutathione (GSH) and enhancing the activities of SOD and catalase (CAT) [51]. Moreover, fish oil was useful in the prevention of depression in old MRL/*lpr* mice through enhancing nuclear erythroid related factor 2 (Nrf2)-mediated antioxidant defense [52].

In brief, *Grewia asiatica* berry juice, *Saccharina japonica*, maqui berry, fish oil, and lycopene mitigated depression by antioxidant action in preclinical models, which should be further studied on human beings.

#### 2.2.3. Modulating the Production of Monoamine Neurotransmitters

The monoamine hypothesis is one of the most generally accepted etiological hypotheses of depression, and modulating monoamine neurotransmitter systems plays an important role in the treatment of depression [53]. Substantial studies have shown that some dietary natural products and nutrients could regulate the production of monoamine neurotransmitters to decrease depression. For instance, deoiled sunflower seeds ameliorated the depression-like behaviors of CUMS-induced mice through upregulating levels of dopamine, serotonin, and norepinephrine [54]. Moreover, another study showed that EGCG improved depression-like behaviors induced by CUMS through reducing the level of serotonin in the colon but increasing it in the hippocampus [55]. Furthermore, a study showed navel orange essential oil exerted anti-depressive effects via increasing serotonin and dopamine levels in the brain [56]. In addition, adzuki bean sprout fermented milk could decrease the symptoms of mild depression by upregulating the expression levels of serotonin, norepinephrine, and dopamine in the hippocampus [57]. Additionally, n-3 PUFA had preventive effects against depression by increasing brainstem serotonin levels and the hippocampal expression of the serotonin-1A receptor [42].

In a word, deoiled sunflower seeds, navel orange essential oil, adzuki bean sprout fermented milk, EGCG, and n-3 PUFA had preventive and therapeutic effects on depression via the modulation of monoamine neurotransmitter production, and clinical trials should be carried out to verify the potential effects and mechanisms of these substances on human beings.

#### 2.2.4. Promoting the Production of Neurotrophins

Neurotrophins are a family of functionally and structurally related proteins, which play a crucial role in promoting the survival, development, and function of neurons, including BDNF, nerve growth factor, neurotrophin-3, and neurotrophin-4 [58]. Several studies have shown that some dietary natural products could increase the production of neurotrophins to improve depression. For instance, garlic essential oil showed an anti-depressive effect against CUMS-induced depression via upregulating the expression levels of hippocampal BDNF, cyclic adenosine monophosphate response element-binding protein (CREB), and protein kinase B [59]. Moreover, *Geum japonicum* is a popular medicinal herb in Asia. It showed neuroprotective effects on corticosterone (CORT)-induced depressive mice by upregulating the expression of BDNF in the hippocampus. It also decreased CORT-induced neurotoxicity in SH-SY5Y cells [60]. In a lupus-prone MRL/*lpr* old mice model of depression, fish oil and conjugated linoleic acid all increased the expression levels of BDNF and synaptic protein in the brain [52]. Additionally, purified anthocyanin from purple cauliflower improved the depressive symptoms of CUMS-induced mice by suppressing the activity of monoamine oxidases and upregulating expression levels of monoamine neurotransmitters, BDNF, and tyrosine kinase receptor B (TrkB) [19]. In a mouse model of CUMS-induced depression, sesamin improved depressive symptoms by increasing neurotrophin expression levels, such as BDNF and neurotrophin-3 [61]. Furthermore, a study found that supplements with probiotics ameliorated the depression-like behaviors of rats with epilepsy through upregulating the expression levels of BDNF and nerve growth factor [62]. In a rat model of male hypogonadism, resveratrol alleviated depression-like behaviors via increasing hippocampal and prefrontal cortical levels of BDNF and neurotrophin-3 [63].

In short, *Geum japonicum*, garlic essential oil, fish oil, conjugated linoleic acid, and anthocyanin could ameliorate depression through increasing the production of BDNF, nerve growth factor, and neurotrophin-3, and their effects and mechanisms should be further investigated by clinical trials.

#### 2.2.5. Inhibition of the HPA Axis Hyperactivity

The HPA axis is an important part of the neuroendocrine system and plays an essential role in the control of stress response and the mediation of mood [64]. The hyperactivity of the HPA axis inhibited the negative feedback signal of cortisol and upregulated the levels of corticotropin-releasing factor (CRF) and adrenocorticotropic hormone (ACTH), which further resulted in depressive symptoms [38]. It was found that eicosapentaenoic acid (EPA)-enriched phospholipids suppressed HPA axis hyperactivity to ameliorate depressive symptoms induced by chronic stress and lipopolysaccharide (LPS) [65]. Additionally, saponin compounds extracted from the traditional Chinese medicine Baihe Zhimu Tang showed an anti-depressive effect through inhibiting the hyperactivation of the HPA axis and improving the synthesis and transport processes of neurotransmitters [66]. In a rat model of post-menopausal depression induced by chronic mild stress and maternal separation, n-3 PUFA exerted antidepressant-like effects through reducing blood levels of CORT and ACTH, decreasing the brain expression of CRF and miRNA-218, and increasing the expression of the glucocorticoid receptor [42]. Furthermore, a study showed n-3 PUFA improved pup separation-induced postpartum depression via regulating the HPA axis by reducing circulating levels of ACTH and CORT and downregulating the expression of hypothalamic CRF [67]. In addition, royal jelly (a common dietary supplement) attenuated CUMS-induced depression by inhibiting the biosynthesis of CORT in the adrenal gland [68].

In brief, royal jelly, EPA-enriched phospholipids, saponin compounds, and n-3 PUFA reduced depressive symptoms by suppressing the hyperactivity of the HPA axis in animal models, which should be certified in clinical trials.

#### 2.2.6. Modulation of Microbiota–Gut–Brain Axis

Nowadays, the important role of gut microbiota in the prevention and management of various diseases has increasingly been recognized [69,70,71,72,73,74,75]. Many studies have shown that disorders of gut microbiota affected the production of cytokines and other inflammatory factors, which regulated several signaling pathways associated with depression [76]. In addition, gut microbiota disorders increased gut barrier permeability and stimulated immune responses and systemic inflammation, which affected the activity and function of the HPA axis and the efficacy of monoamine neurotransmitters [10]. A number of studies have pointed out that several probiotics could improve the symptoms of depression. For example, *Prevotella histicola* was an emerging probiotic and showed a protective effect against estrogen deficiency-induced depression by elevating the abundance of intestinal flora, especially *Akkermansia* and *Lactobacillus* [44]. Moreover, *Lactobacillus casei* exerted anti-depressive effects on postpartum depression via changing the composition of gut microbiota, increasing the expression level of BDNF, and suppressing the activity of the BDNF–MAPK pathway [77]. In addition, a study showed that *Lactobacillus casei* mitigated CUMS-induced depressive symptoms through reversing the structure change of gut microbiota and regulating BDNF/TrkB signaling pathways [78]. Another study showed that *Bifidobacterium* E41 and M2CF22M7 suppressed depression-like behaviors via improving the microbial dysbiosis and enhancing expression levels of serotonin and BDNF [79]. Moreover, *Lactobacillus kefiranofaciens* ZW3 isolated from Tibetan Kefir grains could improve the symptoms of depression through regulating the gut microbiota composition and ameliorating constipation via increasing the content of fecal water [80]. In addition, *Lactobacillus gasseri* NK109 improved *Escherichia coli* K1-induced depression through decreasing the expression level of IL-1α in activated macrophages and regulating gut microbiota via vagus nerve-mediated gut–brain signaling [81].

In addition to the probiotics mentioned above, it was found that other dietary natural products and nutrients could also regulate the gut–brain axis to decrease depression-like behaviors. For example, the high consumption of dietary fiber ameliorated antenatal obesity-induced postpartum depressive-like behaviors, and the mechanisms were related to increasing the expression levels of norepinephrine and serotonin, inhibiting neuroinflammation, promoting the formation of short-chain fatty acids, and the reconstruction of the gut microbiome [82]. In a rat model of ACTH-induced depression, chlorogenic acid exerted anti-depressive effects by increasing the relative abundance of *Burkholderiales* and *Bifidobacterium* and reducing the relative abundance of *Desulfovibrionales* and *Desulfovibrio* [83]. Furthermore, *Bifidobacteria*-fermented red ginseng exerted protective effects against *Escherichia coli*-induced depression by enhancing the abundance of *Bacteroidetes*, decreasing the abundance of *Proteobacteria*, and upregulating the expression of BDNF mediated by NF-κB [84]. In addition, the disorder of gut microbiota was correlated with circadian rhythm disorders, and tea polyphenols were found to regulate the circadian rhythm and increase the abundance of probiotics to attenuate depressive symptoms [10]. Nicotinamide riboside is a form of vitamin B_3_ and is mainly found in milk and yeast [85]. In a mouse model of alcohol-induced depression, nicotinamide riboside changed the composition of gut microbiota, which further decreased the expression levels of inflammation-related cytokines and increased BDNF levels in the hippocampus [86]. Additionally, soy isoflavones improved the depressive symptoms of CUMS rats, and the mechanisms were associated with enhancing the diversity of gut microbiota and upregulating the monoamine neurotransmitters levels [87]. Coniferyl ferulate is a phenolic acid compound mainly found in umbelliferous plants and exerted a protective effect against CUMS-induced depression by improving the reconstruction of the gut microbiome and downregulating the expression levels of IL-6, IL-1β, and TNF-α to decrease colonic inflammation [88]. *Semen sojae praeparatum* is a traditional fermented food and showed anti-depressive effects through modulating the microbiota–gut–brain axis. Specifically, *Semen sojae praeparatum* upregulated the abundance of the genus *Ruminococcaceae_UCG-008* and regulated serotonin, norepinephrine, GABA, and BDNF content in the hippocampus [89].

In a word, some dietary natural products and nutrients could attenuate depression through regulating the microbiota–gut–brain axis in preclinical models, such as *Lactobacillus casei*, *Bifidobacterium* E41 and M2CF22M7, *Lactobacillus kefiranofaciens* ZW3, *Lactobacillus gasseri* NK109, *Prevotella histicola*, *Semen sojae praeparatum, Bifidobacteria*-fermented red ginseng, dietary fiber, tea polyphenols, chlorogenic acid, nicotinamide riboside, soy isoflavones, and coniferyl ferulate. The possible effects and mechanisms of these substances on depression should be further studied in clinical trials.

#### 2.2.7. Other Mechanisms

In addition to the related mechanisms mentioned above, several dietary natural products and nutrients also exerted an anti-depressive effect through other mechanisms. For instance, *Grewia asiatica* berry juice reduced the depression-like behaviors of rats through the modulation of the cholinergic system via decreasing levels of acetylcholinesterase and MDA [50]. In a mouse model of subchronic and mild social defeat stress-induced depression, heat-killed *Lactobacillus helveticus* strain MCC1848 ameliorated depressive symptoms by improving the alteration of gene expression in nervous system development and signal transduction [90]. Furthermore, lotus plumule is the green embryo of lotus seeds and is widely used for tea in China. The alkaloids extracted from lotus plumule mitigated LPS-induced depressive symptoms through suppressing BDNF-mediated endoplasmic reticulum stress and increasing autophagy [91]. In addition, a study found that curcumin attenuated the depression-like behaviors of LPS-induced rats through suppressing excessive synaptic loss and improving synaptic function [92]. Additionally, short-chain fatty acids are the gut microbial metabolites and have neuroprotective bioactivity [93]. A study found that short-chain fatty acids decreased depressive-like behaviors of high fructose-fed mice via inhibiting microglia activation and reducing blood–brain barrier damage [94].

**Table 2:** Effects and mechanisms of dietary natural products and nutrients on depression from experimental studies.

Name	Study Type	Model	Dose	Effects and Mechanisms	Ref.
**Animal Foods**					
Fish oil	In Vivo	Lupus-prone MRL/lpr mice	728 mg/kg	Increased BDNF and synaptic protein Enhanced Nrf2-mediated antioxidant defenses;	[52]
**Plant Foods**					
*Geum japonicum*	In Vivo	ICR mice	30, 100, 300 mg/kg	Exerted neuroprotective effects Upregulated expression of BDNF in hippocampus;	[60]
	In Vitro	SH-SY5Y cells	0, 50, 100 μg/mL	Decreased CORT-induced neurotoxicity;	
Royal jelly	In Vivo	CUMS mice	4.5 g/kg	Attenuated CUMS-induced depression Inhibited the biosynthesis of CORT;	[68]
Purple cauliflower	In Vivo	CUMS mice	50, 100, 200 mg/kg	Improved depressive symptoms Increased content of monoamine neurotransmitter Suppressed activity of MAO Upregulated BDNF, TrkB;	[19]
*Semen sojae praeparatum*	In Vivo	CUMS rats	0.97 g/kg	Exerted antidepressant effect Upregulated Ruminococcaceae_UCG-008 Regulated the 5-HT, NE, GABA, BDNF content	[89]
Deoiled sunflower seeds	In Vivo	CUMS mice	NA	Ameliorated depression-like behaviors Upregulated dopamine, 5-HT, acetylcholine, NE, BDNF;	[54]
*Bifidobacteria*-fermented red ginseng	In Vivo	C57BL/6 mice	10, 25, 50 mg/kg	Exerted protective effects against Escherichia coli-induced depression Enhanced the abundance of Bacteroidetes Reduced the abundance of Proteobacteria Upregulated expression of BDNF;	[84]
Navel orange essential oil	In Vivo	Kunming mice	0.5, 1, 2%	Exerted anti-depressive effects Increased serotonin and dopamine levels in brain;	[56]
Garlic essential oil	In Vivo	CUMS rats	25, 50 mg/kg	Exerted anti-depressive effect Upregulated hippocampal BDNF, CREB, protein kinase B;	[59]
**Beverages**					
Grewia asiatica berry juice	In Vivo	SD male rats	Free access to 5%, 10%, 20%, 30% dilutions	Decreased oxidative damage Increased SOD and GPx levels Modulated the cholinergic system Decreased acetylcholinesterase and MDA levels;	[50]
Maqui berry	In Vivo	Male balb/c mice	25, 50, 100 mg/kg	Ameliorated post-stroke depression Upregulated expression level of GSH Enhanced activities of SOD, CAT;	[51]
Adzuki bean sprout fermented milk	In Vivo	C57BL/6 mice	0.1, 0.2, 0.4 mL/mouse	decreased depressive symptoms Upregulated the expression levels of 5-HT, NE, dopamine;	[57]
**Probiotics**					
*Bifidobacterium* E41 and M2CF22M7	In Vivo	C57BL/6J mice	1 × 10^9^ CFU/mouse	Suppressed depression-like behaviors Improved the gut microbial dysbiosis Enhanced 5-HT and BDNF;	[79]
*Prevotella histicola*	In Vivo	C57 BL/6 mice	1 × 10^9^ CFU/mouse	Protective effect against estrogen deficiency-induced depression Downregulated VCAM, MCP-1, IL-6, IL-8, TNF-α Increased the abundance of Akkermansia and Lactobacillus;	[44]
*Lacticaseibacillus paracasei* NK112	In Vivo	C57 BL/6J mice	NA	Exerted protective effects against Escherichia coli-induced depression Decreased IL-1α, IL-6, TNF-α Inhibited the activity of NF-κB;	[45]
*Lactobacillus casei*	In Vivo	CUMS rats	NA	Mitigated depressive symptoms Reversed the structure change of gut microbiota Regulated BDNF/TrkB signaling;	[78]
*Lactobacillus casei*	In Vivo	Pregnant rats	8 × 10^8^ CFU/kg	Exerted anti-depressive effects Changed the composition of gut microbiota Increased expression level of BDNF Suppressed BDNF–MAPK pathway	[77]
*Lactobacillus gasseri* NK109	IN VIVO	Mice	1 × 10^8^, 1 × 10^9^ CFU/mouse	Improved Escherichia coli K1-induced depression Decreased the expression level of IL-1α Regulated gut microbiota;	[81]
*Lactobacillus kefiranofaciens* ZW3	In Vivo	CUMS mice	1 × 10^7^, 1 × 10^8^, 1 × 10^9^ CFU/mouse	Improved the symptoms of depression Regulated the gut microbiota composition Ameliorated constipation;	[80]
*Lactobacillus helveticus strain* MCC1848	In Vivo	C57BL/6J mice	1 × 10^11^ CFU/mouse	Ameliorated depressive symptoms Improved the alteration of gene expression in nervous system development and signal transduction;	[90]
**Nutrients**					
SCFAs	In Vivo	High fructose-fed mice	NA	Decreased depression-like behaviors Inhibited microglia activation Reduced blood–brain barrier damage;	[94]
n-3 PUFA	In Vivo	Wistar rats	0 en% of n-3 PUFA, 1 en% of n-3 PUFA	Ameliorated post-menopausal depression Increased brainstem serotonin level Increased serotonin-1A receptor, BDNF, CREB, miRNA-155, GR Downregulated IL-6, IL-1β, TNF-α, prostaglandin E-2, CORT, ACTH, CRF, miRNA-218;	[42]
n-3 PUFA	In Vivo	Wistar rats	0 en% of n-3 PUFA, 1 en% of n-3 PUFA	Regulated the HPA axis Reduced circulating levels of ACTH and CORT Downregulated expression of CRF;	[67]
EGCG	In Vivo	CUMS rats	50 mg/kg	Exerted anti-depressive effects Reduced IL-6 and NO Decreased caspase-3 and caspase-9;	[46]
EGCG	In Vivo	CUMS rats	50 mg/kg	Improved depression-like behaviors Reduced serotonin in the colon Increased serotonin in the hippocampus;	[55]
EPA-PL	In Vivo	ICR mice	NA	Ameliorated depressive symptoms Suppressed HPA axis hyperactivity;	[65]
Sesamin	In Vivo	CUMS mice	50 mg/kg	Improved depressive symptoms Increased BDNF, NT-3;	[61]
Resveratrol	In Vivo	Wistar-Kyoto male rats	40 mg/kg	alleviated depression-like behaviors increased BDNF, NT3;	[63]
Dietary fiber	In Vivo	C57BL/6J mice	NA	Increased the expression levels of NE, 5-HT Inhibited neuroinflammation Promoted formation of short-chain fatty acids and reconstruction of gut microbiome;	[82]
Dietary pectins	In Vivo	BALB/c mice	50 mg/kg	Suppressed depression-like behaviors Increased the levels of IL-6, IFN-γ Downregulated the protein level of STAT3;	[95]
Coniferyl ferulate	In Vivo	C57BL/6 SPF mice	50 mg/kg	Exerted protective effect Improved the reconstruction of gut microbiome Downregulated the expression levels of IL-6, IL-1β, TNF-α;	[88]
Coniferyl ferulate	In Vitro	PC12 cells	0.2, 2, 20 μmol/L	Exerted anti-depressive effect Decreased the production of ROS Suppressed mitochondrial apoptotic pathways;	[96]
Nicotinamide riboside	In Vivo	C57BL/6J mice	400 mg/kg	Changed the composition of gut microbiota Decreased inflammation-related cytokines Increased BDNF levels;	[86]
Soy isoflavones	In Vivo	CUMS rats	40, 80, 160 mg/kg	Improved depressive symptoms Enhanced the diversity of gut microbiota Upregulated monoamine neurotransmitter levels;	[87]
Curcumin	In Vivo	Wistar male rats	40 mg/kg	Attenuated depression-like behaviors Suppressed excessive synaptic loss Improved synaptic function;	[92]
Lycopene	In Vitro	SH-SY5Y cells	1~10 μM	Alleviated oxidative damage Reduced 8-OHdG, MDA, and protein carbonyls expressions Inhibited PERK signaling pathway;	[48]
Chlorogenic acid	In Vivo	Wistar rats	500 mg/kg	Had anti-depressive effects Increased Burkholderiales, Bifidobacterium Reduced Desulfovibrionales, Desulfovibrio;	[83]
Apple phenolic	In Vivo	Kunming mice	200 ppm in normal saline	Improved lead acetate-induced depression-like behaviors Attenuated neuroinflammation and neuronal apoptosis Regulated miR-22-3p/SIRT1 signaling pathway;	[39]
Alkaloids	In Vivo	C57BL/6N mice	200 mg/kg	Mitigated LPS-induced depressive symptoms Inhibited neuroinflammation Repressed BDNF-mediated endoplasmic reticulum stress Increased autophagy;	[91]
	In Vitro	BV2 cells	50 μg/mL	Inhibited pro-inflammatory mediators and NO production;	
Aponin compounds	In Vivo	CMS rats	240 mg/kg	Exerted anti-depressive effect Inhibited the hyperactivation of HPA axis Improved the synthesis and transport processes of neurotransmitters;	[66]
*Saccharina japonica* ethanol extract	In Vivo	C57BL/6 mice	1, 2, 4 g/kg	Decreased the depression-like behaviors Increased activity of superoxide dismutase Increased anti-inflammatory cytokines Downregulated NF-κB, NOD-like receptor 3, TLR-4;	[43]

ACTH, adrenocorticotropic hormone; BDNF, brain-derived neurotrophic factor; CAT, catalase; CORT, corticosterone; CREB, cyclic adenosine monophosphate response element-binding protein; CRF, corticotropin-releasing factor; CMS, chronic mild stress; CUMS, chronic unpredictable mild stress; EGCG, Epigallocatechin gallate; en%, energy percent; GABA, gamma-aminobutyric acid; GPx, glutathione peroxidase; GR, glucocorticoid receptor; GSH, glutathione; 5-HT, serotonin; IL, interleukin; LPS, lipopolysaccharide; MAO, monoamine oxidases; MAPK, mitogen-activated protein kinase; MCP-1, macrophage chemoattractant protein 1; MDA, malondialdehyde; NA, not available; NE, norepinephrine; NF-κB, nuclear factor kappa-B; NO, nitric oxide; Nrf2, nuclear erythroid related factor 2; NT-3, neurotrophin-3; 8-OHdG, 8-hydroxydeoxyguanosine; PERK, protein kinase-like endoplasmic reticulum kinase; PUFA, polyunsaturated fatty acids; SCFAs, short-chain fatty acids; SD, Sprague Dawley; SIRT1, Sirtuin 1; TLR-4, Toll-like receptor-4; TNF-α, tumor necrosis factor-α; TrkB, tyrosine kinase receptor B; VCAM, vascular cell adhesion molecule.

In short, *Grewia asiatica* berry juice, heat-killed *Lactobacillus helveticus* strain MCC1848, alkaloids from lotus plumule, and curcumin could ameliorate depression, and the potential mechanisms included the modulation of the cholinergic system, suppression of mitochondrial apoptotic pathways, inhibition of BDNF-mediated endoplasmic reticulum stress, improvement of synaptic function, and promotion of nervous system development and signal transduction. In the future, these mechanisms should be widely studied, and also should be verified based on human beings.

### 2.3. Clinical Trials

A placebo-controlled and randomized study of 25 healthy college students showed that daily drinking apple cider vinegar could reduce the risk of depression, which might be related to inhibit activation of hexosamine pathway and promote metabolism of glycine, serine and threonine [97]. Another double-blind, randomized and controlled clinical trial of 60 women with depressive disorder found that supplement with flaxseed oil twice a day for 10 weeks increased the concentration of serum BDNF and attenuated depressive symptoms [98]. In addition, a randomized controlled pilot study of 143 postpartum women showed that frequently drinking magnolia tea had protective effects against postpartum depression [99]. In a randomized double-blind and placebo-controlled trial of 64 healthy adolescents, it was found that daily supplement with wild blueberry had positive effects on the prevention of depression [100]. Furthermore, a randomized, double-blind, placebo-controlled trial of 82 patients with depression showed that the consumption of *Lacticaseibacillus paracasei* strain Shirota for 9 weeks improved depression-like behaviors and ameliorated constipation of patients, which was related to upregulate the beneficial *Adlercreutzia*, *Megasphaera* and *Veillonella* levels [101]. In a randomized trial of 24 healthy volunteers, consumption of yogurt twice daily after lunch and dinner did not significantly change biomarker levels of depression, but when the participants regularly consumed yogurt and daily exercised, the level of serotonin increased [102]. Additionally, a meta-analysis of 36 clinical trials including 2788 participants showed that the higher flavonoid consumption improved depressive symptoms (Mean difference = −1.65; 95% CI, −2.54, −0.77) [103]. Moreover, a meta-analysis of 23 randomized controlled trials including 237 participants revealed the saffron improved the symptoms of depression, compared with placebo [104].

In brief, clinical trials have found that saffron, wild blueberry, flaxseed oil, magnolia tea, apple cider vinegar, *Lacticaseibacillus paracasei* strain Shirota and flavonoid could decrease depression. Although these results from clinical trials are more reliable than those from preclinical studies, more randomized, double-blind, placebo-controlled trials should be carried out for verifying their effects on different race persons in different places of world.

## 3. Anxiety

Anxiety is a common psychiatric disease, and the oxidative stress, inflammation, noradrenergic and serotonergic dysfunction are responsible for its initiation and development [13]. Accumulating evidence has supported that dietary natural products and nutrients could decrease the risk of anxiety through different mechanisms.

### 3.1. Epidemiological Studies

A lot of population-based epidemiological studies have found that some dietary natural products and nutrients could reduce the risk of anxiety (Table 3). For example, a cross-sectional study found that legume and nut consumption was negatively associated with the risk of anxiety in men (OR, 0.34; 95% CI, 0.14–0.82), but not in women (OR, 1.06; 95% CI, 0.63–1.77) [35]. The results of the gender difference might be due to the different effects of gonadal hormones on anxiety in men and women. A cross-sectional study of 3175 Iranian adults showed that higher intake of branched-chain amino acids was inversely associated with incidence of anxiety [105]. In addition, the consumption of <8.1 g/day dietary fiber increased the incidence of depression (OR, 2.757; 95% CI, 1.035–7.346), compared with those who consumed ≥15.4 g/day [26]. A cross-sectional study of 3362 adults found that higher consumption of vitamin B_6_ reduced the risk of anxiety in women, but not in men [106]. The results of the gender difference also could be from the difference of sex hormones. Additionally, it was found that higher intake of n-3 fatty acids daily was associated with a lower incidence of anxiety in a cross-sectional study [107]. A meta-analysis including 17 cross-sectional studies showed that those who consumed meat was associated with lower risk of anxiety, compared with those who didn’t consumed meat (Hedges’ g, 0.17; 95% CI, 0.03–0.31) [108]. However, a cross-sectional study showed inconsistent results, which found that those who consumed >25 g sugar/day from soft drinks were associated with higher risk of anxiety, compared with those who did not drink soft drinks [36]. This result was consistent with that of depression mentioned above.

In summary, many epidemiological evidence supported that some dietary natural products had positive effects against anxiety, such as dietary fiber, n-3 fatty acids, vitamin B_6_, legume and nut. However, drinking too much soft drinks could increase the risk of anxiety, which suggested we should reduce the consumption of soft drinks. The results from epidemiological studies are only indicative. It needs cell and animal experiments to investigate the possible effects and mechanisms of these substances on anxiety, and clinical trials should be carried out to verify effects on human beings.

### 3.2. Experimental Studies

**Figure 2:**
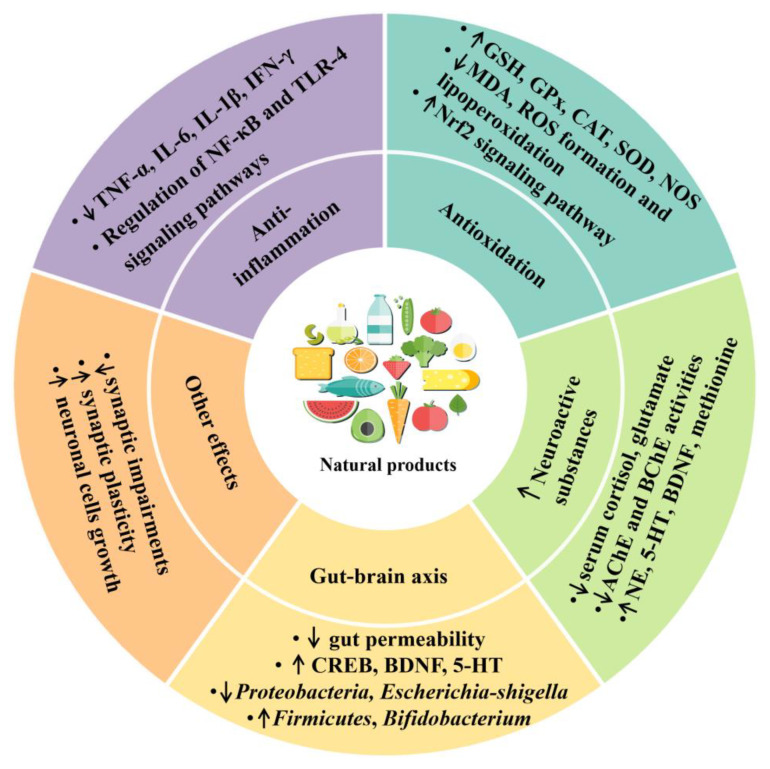
The effects and mechanisms of dietary natural products on anxiety. ↑ represents increase, and ↓ represents decrease. AChE, acetylcholinesterase; BChE, butyrylcholinesterase; BDNF, brain-derived neurotrophic factor; CAT, catalase; CREB, cyclic adenosine monophosphate response element-binding protein; GPx, glutathione peroxidase; GSH, glutathione; 5-HT, serotonin; IFN-γ, interferon-gamma; IL, interleukin; MDA, malondialdehyde; NE, norepinephrine; NF-κB, nuclear factor kappa-B; NOS, nitric oxide synthase; Nrf2, nuclear erythroid related factor 2; ROS, reactive oxygen species; SOD, superoxide dismutase; TLR-4, Toll-like receptor-4; TNF-α, tumor necrosis factor-α.

A number of studies have pointed out that some dietary natural products and nutrients had protective effects against anxiety, and the underlying mechanisms of action were mainly involved in anti-inflammation, antioxidation, neuroprotection and regulation of gut–brain axis [11], which would be discussed in detail below (Table 4 and Figure 2).

#### 3.2.1. Anti-Inflammatory Effect

Neuroinflammation is correlated with the progression of anxiety, and many dietary natural products and nutrients have anti-inflammatory bioactivity to reduce anxiety-related symptoms. For example, sesamol is a liposoluble lignan isolated from sesame products and an in vivo study found that it reduced anxiety-like behaviors of mice with inflammatory bowel disease through decreasing neuroinflammatory responses via inhibition of TLR-4/NF-κB pathway [109]. Furthermore, in a rat model of LPS-induced anxiety, honey had protective effects against anxiety by decreasing the expression levels of TNF-α and IL-6 [20]. Additionally, the combination of caffeine and caffeic acid attenuated anxiety-like behaviors of mice with LPS-induced neuroinflammation through decreasing inflammatory marker levels [110]. Moreover, bergamot essential oil could relieve anxiety-like behaviors of aluminum trichloride-exposed rats via downregulating the levels of IL-1β, IL-6 and TNF-α in the hippocampus and the frontal cortex [111]. Furthermore, in a cafeteria diet-induced obese rat model, n-3 PUFA attenuated the symptoms of obesity-induced anxiety via exerting anti-inflammatory effects by decreasing IL-6 levels in the liver and TNF-α levels in the brain [112]. In addition, a red pomegranate fruit extract-based formula ameliorated anxiety-like behaviors through reducing the levels of serum inflammatory cytokines NF-κB, TNF-α, IL-6, IL-1β and IFN-γ [113].

In brief, honey, coffee, bergamot essential oil, sesamol and n-3 PUFA could reduce the symptoms of anxiety in animal models due to their anti-inflammatory activity, which should be certified in clinical trials.

#### 3.2.2. Antioxidant Effect

The brain is vulnerable to oxidative stress because oxidative stress can affect the function of neurotransmitters and disrupt the integrity of meninges, which could lead to anxiety [114]. Some dietary natural products and nutrients with antioxidant activity have been widely studied for prevention and management of anxiety. For instance, goat milk is a good source of fat acids and an in vivo study showed goat milk fats palliated anxiety-like behaviors of rats by decreasing MDA levels and increasing GSH levels in the brain [114]. In a mouse model of dextran sulfate sodium-induced inflammatory bowel disease, sesamol attenuated anxiety-like behaviors by stimulating the Nrf2 antioxidant signaling pathway [109]. Additionally, tangeretin decreased anxiety-like behaviors of rats with post-traumatic stress disorder, which was associated with promoting the activation of Nrf2 signaling pathway [115]. Moreover, it was found that bergamot essential oil improved symptoms of stress-induced anxiety by enhancing the activities of GPx, CAT and SOD in the hippocampus and the frontal cortex [111]. Furthermore, *Blumea lacera* is an edible plant with various medicinal values and its leaf methanol extract mitigated anxiety-related behaviors by suppression of ROS formation via redox-related signaling pathway [116]. In a high fat diet-induced obese mouse model, low dose of alcohol decreased anxiety-like behaviors through upregulating the expression level of adiponectin and promoting the activation of Nrf2 signaling pathway [117]. Moreover, it was found that a red pomegranate fruit extract-based formula could improve the anxiety-like behaviors via decreasing the level of MDA and promoting the activities of nitric oxide synthase, SOD and CAT [113]. In addition, chamomile decoction ameliorated high fat diet-induced anxiety of rats through suppression of lipoperoxidation, and promoting antioxidant enzyme activities of SOD, CAT and GPx [118].

In short, *Blumea lacera,* goat milk fat, bergamot essential oil, chamomile decoction, sesamol and tangeretin had preventive and therapeutic effects on anxiety through antioxidant action in preclinical models, and the studies based on human beings should be conducted to verify their effect and mechanism.

#### 3.2.3. Modulation of Gut Microbiota

The gut microbiota can regulate the neural and immune systems by the gut–brain axis, and improving gut microbiota dysbiosis could have an important effect on anxiety [119]. It was found that *Lactococcus lactis* WHH2078 could improve the CUMS-induced anxiety symptoms through regulation of serotonin metabolism and gut microbiome composition. Specifically, it decreased the serum CORT level, increased the central levels of serotonin and BDNF, and restored abundances of *Firmicutes* and *Bacteroidetes* [120]. Moreover, *Lactobacillus sakei* reduced high fat diet-induced anxiety-like behaviors through inhibiting the population of *Proteobacteria* and decreasing fecal LPS level [121]. In a mouse model of streptomycin-induced dysbiosis, *Escherichia coli Nissle* 1917 attenuated anxiety-like behaviors of mice through inhibiting the pathologic gut–brain circuit [122]. In addition, *Pediococcus acidilactici* CCFM6432 mitigated chronic stress-induced anxiety symptoms via modulation of gut–brain axis, which was associated with suppressing the pathogenic bacteria (such as *Escherichia-shigella*), improving beneficial bacteria growth (such as *Bifidobacterium*), inhibiting hyperactivity of HPA axis and upregulating the expression level of CREB in hippocampus [123]. Additionally, in a mouse model of ulcerative colitis and chronic stress, *Weissella paramesenteroides* WpK4 decreased anxiety-related behaviors by reducing gut permeability and regulating gut–brain axis [124].

In short, *Lactobacillus sakei*, *Lactococcus lactis* WHH2078, *Escherichia coli Nissle* 1917, *Pediococcus acidilactici* CCFM6432 and *Weissella paramesenteroides* WpK4 could ameliorate anxiety via regulating gut microbiota. In the future, the effect of more probiotics and prebiotics on anxiety should be investigated through targeting gut microbiota, and should be verified by clinical trials.

#### 3.2.4. Regulation of Production of Neuroactive Substances

A great number of evidence has supported that dysfunction of neurotransmitter systems would result in impairment of intracellular signal processing, which was involved in the neurobiological processes of anxiety [125]. It was found that supplement with sesamol improved anxiety induced by inflammatory bowel disease through repairing synaptic impairments, upregulating the expression levels of norepinephrine and serotonin, increasing BDNF levels via the BDNF/TrkB/CREB signaling pathway [109]. Furthermore, breadfruit pulp exerted protective effects against anxiety in zebrafish by regulating the serotoninergic system [126]. Additionally, panaxynol mainly exists in the umbelliferae plants and it was found to improve CUMS-induced anxiety symptoms in mice by suppressing the HPA axis hyperfunction, increasing the release of hippocampal serotonin, and promoting synaptic plasticity in the hippocampus [127]. Moreover, a red pomegranate fruit extract-based formula exerted neuroprotective effects on anxiety through increasing the level of serotonin in hippocampus, which was associated with suppressing the activity of indoleamine-2,3-dioxygenase and improving the activity of tryptophan hydroxylase [113]. In a rat model of sub-chronic stress-induced anxiety, saffron had protective effects on anxiety via downregulating serum cortisol level and upregulating the gene expression of BDNF in hippocampus [128]. In addition, treatment with 0.4 mg/kg/day L-theanine attenuated anxiety-related behaviors of rats, which was correlated with downregulating glutamate level and upregulating methionine level in the brain to improve hippocampal activity [129]. Furthermore, in a high fat diet-induced obese rat model, chamomile decoction treatment had effects on reducing anxiety-like behaviors via inhibiting the activities of acetylcholinesterase and butyrylcholinesterase [118].

In a word, breadfruit pulp, chamomile decoction, saffron, L-theanine, sesamol and panaxynol could improve anxiety through modulating the production of neuroactive substances, and clinical trials should be performed to certify the possible effects and mechanisms of these substances on human beings.

#### 3.2.5. Other Mechanisms

Except for the mechanisms mentioned above, several dietary natural products and nutrients also have neuroprotective effect against anxiety via other mechanisms. For example, queen bee acid is the main fatty acid of royal jelly and it had effects on decreasing anxiety-like behaviors and promoting the growth of neurons in aged Sprague Dawley rats [130]. Furthermore, low dose of curcumin treatment could exert anxiolytic effect through improving the synaptic plasticity to enhance neural circuits, but high dose of curcumin reversed anxiolytic effect because of inducing neuroinflammation to affect hippocampal neurogenesis [131]. This also could be because antioxidants with low dose would show antioxidant activity in vivo, but they with high dose would exhibit pro-oxidative activity in vivo [132,133,134]. Additionally, in a cadmium-exposed mouse model, curcumin decreased anxiety-like behaviors by promoting the production of viable prefrontal cortex neuronal cells, and suppressing neuroinflammation in prefrontal cortex [135]. In addition, the intermediate dose of blackberry juice decreased anxiety-like behaviors, and the anxiolytic mechanism might be similar to diazepam [136].

**Table 4:** Effects and mechanisms of dietary natural products and nutrients on anxiety from experimental studies.

Name	Study Type	Model	Dose	Effects and Mechanisms	Ref.
**Animal foods**					
Honey	In Vivo	Wistar rats	0.26, 0.31, 0.36 g/kg	Had protective effects against anxiety Decreased TNF-α, IL-6;	[20]
**Plant foods**					
Saffron	In Vivo	Rats	30, 60 mg/kg	Exerted anxiolytic effect Downregulated serum cortisol level Upregulated BDNF in hippocampal;	[128]
Bergamot essential oil	In Vivo	SD rats	200 mg/kg	Improved anxiety Decreased IL-1β, IL-6, TNF-α Enhanced the activity of GPx, CAT, SOD;	[111]
Red pomegranate fruit extract-based formula	In Vivo	C57BL/6J mice	2.0, 1.5, 1.0 mg/g	Exerted anxiolytic effect Increased 5-HT in hippocampus Suppressed indoleamine-2,3-dioxygenase Improved tryptophan hydroxylase Reduced NF-κ B, TNF-α, IL-6, IL-1β, IFN-γ, MDA Promoted the activities of NOS, SOD and CAT;	[113]
**Beverages**					
Low-dose alcohol	In Vivo	C57BL/6 mice	0.8 g/kg	Decreased anxiety-like behaviors Upregulated adiponectin Activated Nrf2 signaling pathway;	[117]
Breadfruit pulp	In Vivo	Zebrafish	NA	Exerted anxiolytic effect Regulated the serotoninergic system	[126]
**Probiotics**					
*Lactobacillus sakei*	In Vivo	Mice	OK67: 2 × 10^8^, 1 × 10^9^, 2 × 10^9^ CFU/mouse PK16: 1 × 10^9^, 5 × 10^9^ CFU/mouse	Mitigated anxiety-like behaviors Inhibited the population of *Proteobacteria* Decreased fecal lipopolysaccharide levels Inhibited NF-κB Increased AMPK;	[121]
*Lactococcus lactis* WHH2078	In Vivo	CUMS mice	1 × 10^9^ CFU/mouse	Decreased serum CORT Increased 5-HT, BDNF Restored abundances of *Firmicutes* and *Bacteroidetes*;	[120]
*Escherichia coli* Nissle 1917	In Vivo	C57BL/6 mice	0.5 × 10^10^, 1 × 10^10^ CFU/mouse	Attenuated anxiety-like behaviors Inhibited the pathologic gut–brain circuit;	[122]
*Weissella paramesenteroides* WpK4	In Vivo	C57BL/6 mice	1 × 10^8^ CFU/mouse	Mitigated anxiety-related behaviors Decreased gut permeability Regulation of gut–brain axis;	[124]
*Pediococcus acidilactici CCFM6432*	In Vivo	C57BL/6 mice	5 × 10^9^ CFU/mouse	Attenuated anxiety-like behaviors Improved the gut microbial composition Inhibited hyperactivity of HPA axis Upregulated phosphorylated CREB;	[123]
**Nutrients**					
Caffeine and caffeic acid	In Vivo	Swiss albino mice	Caffeine: 15 mg/kg Caffeine + caffeic acid: 10 mg/kg + 5 mg/kg	Attenuated anxiety-like behaviors Decreased inflammatory markers levels;	[110]
Sesamol	In Vivo	C57BL/6J mice	100 mg/kg	Reduced anxiety-like behaviors Decreased neuroinflammatory responses Inhibition of TLR-4/NF-κB pathway Stimulated Nrf2 signaling pathway Increased BDNF, NE, 5-HT Repaired synaptic impairments Regulated BDNF/TrkB/CREB signaling pathway;	[109]
Curcumin	In Vivo	Rats	0.1, 0.5%	Low doses: exerted anxiolytic effect, improved the synaptic plasticity, enhanced neural circuits High doses: reversed anxiolytic effect, induced neuroinflammation;	[131]
Curcumin	In Vivo	Swiss albino mice	20, 40, 80, 160 mg/kg	Ameliorated anxiety-like behaviors Promoted the production of neuronal cells Suppressed neuroinflammation;	[135]
n-3 PUFA	In Vivo	Wistar rats	500 mg/kg	Attenuated obesity-induced anxiety Exerted anti-inflammatory effect Decreased IL-6, TNF-α;	[112]
*Blumea lacera* leaf methanol extract	In Vivo	Swiss albino mice	200, 400 mg/kg	Mitigated anxiety-like behaviors Suppressed ROS formation;	[116]
Chamomile decoction	In Vivo	Wistar rats	100 mg/kg	Ameliorated high fat diet-induced anxiety Suppressed lipoperoxidation Promoted antioxidant enzymes activities Inhibited AChE, BChE;	[118]
Goat milk fat	In Vivo	Wistar rats	NA	Palliated anxiety-like behaviors Decreased MDA Increased GSH;	[114]
Tangeretin	In Vivo	Rats	100, 200 mg/kg	Decreased anxiety-like behaviors Activated Nrf2;	[115]
Panaxynol	In Vivo	CUMS mice	1.0 mg/kg	Improved CUMS-induced anxiety symptoms Suppressed the HPA axis hyperfunction Increased the release of 5-HT Promoted synaptic plasticity;	[127]
L-theanine	In Vivo	WKY rats	0.4 mg/kg	Attenuated anxiety-related behaviors Decreased glutamate levels Increased methionine levels Improved hippocampal activity;	[129]
Queen bee acid	In Vivo	SD rats	12, 24 mg/kg	Decreased anxiety-like behaviors Promoted the growth of neurons;	[130]

AChE, acetylcholinesterase; AMPK, AMP-activated protein kinase; BChE, butyrylcholinesterase; BDNF, brain-derived neurotrophic factor; CAT, catalase; CORT, corticosterone; CREB, cyclic adenosine monophosphate response element-binding protein; CUMS, chronic unpredictable mild stress; GABA, gamma-aminobutyric acid; GPx, glutathione peroxidase; GSH, glutathione; HPA, hypothalamic-pituitary-adrenal; 5-HT, serotonin; IFN-γ, interferon-gamma; IL, interleukin; MCP-1, macrophage chemoattractant protein 1; MDA, malondialdehyde; NA, not available; NE, norepinephrine; NF-κ B, nuclear factor kappa-B; NOS, nitric oxide synthase; Nrf2, nuclear erythroid related factor 2; OK67, heat-labile Lactobacillus sakei OK67; PK16, heat-stable Lactobacillus sakei PK16; PUFA, polyunsaturated fatty acids; SD, Sprague Dawley; SOD, superoxide dismutase; TLR-4, Toll-like receptor-4; TNF-α, tumor necrosis factor-α; TrkB, tyrosine kinase receptor B.

In brief, blackberry juice, queen bee acid and curcumin could attenuate the symptoms of anxiety, and the underlying mechanisms were associated with promoting the growth of neurons and improving the synaptic plasticity. In the future, more mechanisms of dietary natural products and nutrients on anxiety should be investigated, and also should be verified based on human beings.

### 3.3. Clinical Trials

In a double-blind, randomized, and placebo-controlled trial of 58 patients with generalized anxiety disorder, probiotics as adjunctive therapy for 8 weeks had beneficial effects on improving anxiety symptoms [137]. Another randomized, double-blind, and placebo-controlled study of 103 participants found that *Lactobacillus plantarum* P8 attenuated anxiety-like symptoms, which was associated with downregulating the expression levels of pro-inflammatory cytokines [138]. Additionally, a meta-analysis of seven clinical trials showed the higher consumption of flavonoids (≥50 mg/day) could improve the symptoms of anxiety (Hedges’ g,−0.741; 95% CI, −1.266, −0.217) [13]. Moreover, a meta-analysis of 23 randomized controlled trials revealed that saffron had positive effects on improving the symptoms of anxiety (Hedges’ g, 0.95; 95% CI, 0.27–1.63) [104]. In addition, a prospective, mixed, and experimental pilot study of 51 patients with multiple sclerosis showed that the combination treatment of 800 mg/day EGCG and 60 mL/day coconut oil had a protective effect against anxiety [139]. However, in a double-blind, randomized, and placebo-controlled trial of 46 participants with generalized anxiety disorder, a supplement with L-theanine could not exert significant effects on anxiety-like behaviors [140]. Additionally, a meta-analysis of 12 randomized, double-blind, placebo-controlled trials showed that a supplement with B-group vitamins had no benefit for the symptoms of anxiety (standardized mean difference, 0.03; 95% CI, −0.13, 0.20) [141].

In a word, clinical trials showed that *Lactobacillus plantarum* P8, flavonoids, saffron, EGCG, and coconut oil attenuated anxiety, but L-theanine and B-group vitamins had no effect on anxiety. It should be pointed out that the results regarding L-theanine and B-group vitamins from clinical trials were different to those from epidemiological and experimental studies, where vitamin B_6_ and L-theanine showed anxiolytic effects [106,129]. This also suggests that the results from the epidemiological and experiment studies should be further verified in clinical trials.

## 4. Conclusions and Perspectives

Depression and anxiety are the most common neuropsychiatric diseases, which result in a decrease in the quality of life of patients across the world. Epidemiological studies showed that some dietary natural products and nutrients had protective effects against depression, such as fish, walnuts, coffee, tea, carbohydrates, dietary fiber, and selenium. Moreover, dietary fiber, vitamin B_6_, branched-chain amino acids, and n-3 fatty acids could reduce the risk of anxiety. However, drinking too much soft drink could increase the risk of both depression and anxiety. Experimental studies found that many dietary natural products and nutrients exerted anti-depressive effects through different mechanisms, including the inhibition of inflammation, amelioration of oxidative stress, regulation of the level of monoamine neurotransmitters and BDNF, and modulation of the gut–brain axis and the HPA axis. Furthermore, some dietary natural products and nutrients had protective effects on anxiety through different mechanisms, such as suppressing inflammation and oxidative stress, modulating the microbiota–gut–brain axis, and increasing expression levels of neuroactive substances. Clinical trials also revealed that some dietary natural products and nutrients could be beneficial for the prevention and treatment of these diseases, such as saffron, wild blueberries, flaxseed oil, magnolia tea, apple cider vinegar, *Lacticaseibacillus paracasei* strain Shirota, and flavonoids for depression, and *Lactobacillus plantarum* P8, flavonoids, saffron, EGCG, and coconut oil for anxiety, but L-theanine and B-group vitamins had no effect on anxiety. In the future, the effects of more dietary natural products and nutrients on these diseases should be evaluated, and the underlying mechanisms should be studied more comprehensively. It should be pointed out that this is a narrative review, and the possible effects and mechanisms of dietary natural products and nutrients on depression and anxiety are summarized and concluded. Because most of the results come from preclinical models, more clinical trials should be carried out to verify these potential effects and the mechanisms of dietary natural products and nutrients on depression and anxiety in human beings. Furthermore, their adverse effects should also be paid attention to. In addition, some dietary natural products and nutrients with anti-depressive and anxiolytic effects could be developed into functional foods to prevent and manage these diseases.

## Figures and Tables

**Table 1:** Effects of dietary natural products and nutrients on depression from epidemiological studies.

Name	Study Type	Participants	Dose	Effects	Ref.
**Animal Foods**					
Fish	Cross-sectional study	9183 Korean adults 19 ≤ age ≤ 64	4 times/week vs. <1 time/week	Associated with a lower risk of depression Total: OR, 0.52; 95% CI, 0.37–0.74 Men: OR, 0.64; 95% CI, 0.30–1.37 Women: OR, 0.44; 95% CI, 0.29–0.67;	[31]
Fatty fish	Cross-sectional study	6587 participants	Second, third, fourth quintiles vs. the lowest quintiles	U-shaped relationship with depression (OR, 0.77; 95% CI, 0.63–0.94) (OR, 0.71; 95% CI, 0.58–0.87) (OR, 0.78; 95% CI, 0.64–0.96);	[32]
**Plant foods**					
Legume and nut	Cross-sectional study	3172 participants 18 ≤ age ≤ 55	The highest vs. the lowest quartile	No association with depression Men: OR, 0.96; 95% CI, 0.54–1.71 Women: OR, 0.98; 95% CI, 0.65–1.48;	[35]
Walnut	Cross-sectional study	26,656 participants	Daily walnut consumption vs. no nut consumption	Protective effect on depression Total: OR, 0.67; 95% CI, 0.48–0.93 Men: OR, 0.72; 95% CI, 0.41–1.27 Women: OR, 0.62; 95% CI, 0.46–0.84;	[30]
Seaweed and mushroom fiber	Cross-sectional study	2960 adults 19 ≤ age ≤ 64	Seaweed fiber: ≥1.02 g/day vs. <0.31 g/day Mushroom fiber: ≥0.14 g/day vs. <0.03 g/day	Inversely associated with depressive symptoms Seaweed fiber: OR, 0.38; 95% CI, 0.20–0.72 Mushroom fiber: OR, 0.18; 95% CI, 0.01–0.37;	[29]
**Beverages**					
Green tea	Cross-sectional study	9576 Korean adults age ≥ 19	≥3 cups/week vs. none or <1 cup/week	Decreased the prevalence of depression (OR, 0.79; 95% CI, 0.63–0.99);	[25]
Tea	Cohort study	3177 participants age ≥ 55	≥3 cups/day vs. none or <1 cup/day	Associated with a lower risk of depression (OR, 0.32; 95% CI, 0.12–0.84);	[24]
Coffee	Cohort study	14,413 university graduates	≥4 cups/day vs. <1 cup/day	Associated with a lower risk of depression (HR, 0.37; 95% CI, 0.15–0.95);	[33]
Soft drink	Cross-sectional study	8085 college students	>25 g sugar/day from soft drinks vs. none	Associated with a higher risk of depression (Mean difference, 0.22; 95% CI, 0.15–0.29);	[36]
Sugar-sweetened drink	Cohort study	15,546 Spanish university graduates	The highest vs. the lowest quartile	No association with depression (HR, 1.12; 95% CI, 0.90–1.41);	[37]
**Nutrients**					
Carbohydrate	Two-sample Mendelian randomization analysis	268,922 samples	NA	A causal relationship with a lower risk of depression (OR, 0.42; 95% CI, 0.28–0.62);	[34]
Dietary fiber	Cross-sectional study	459 hypertensive patients	10.5–15.4 g/day vs. ≥15.4 g/day	Associated with a higher incidence of depression (OR, 2.641; 95% CI, 1.050–6.640);	[26]
Selenium	Cross-sectional study	736 Brazilian farmers 18 ≤ age ≤ 59	≥95.26 μg/day vs. ≤66.66 μg/day	Decreased the risk of depression (OR, 0.461; 95% CI, 0.236–0.901);	[27]

CI, confidence interval; NA, not available; OR, odds ratio; HR, hazard ratios.

**Table 3:** Effects of dietary natural products and nutrients on anxiety from epidemiological studies.

Name	Study Type	Participants	Dose	Effects	Ref.
**Plant foods**					
Legume and nut	Cross-sectional study	3172 participants 18 ≤ age ≤ 55	The highest vs. the lowest quartile	Protective effect on anxiety in men Men: OR, 0.34; 95% CI, 0.14–0.82 Women: OR, 1.06; 95% CI, 0.63–1.77;	[35]
**Beverages**					
Soft drinks	Cross-sectional study	8085 college students	>25 g sugar/day from soft drinks vs. none	Associated with a higher risk of anxiety (Mean difference, 0.11; 95% CI, 0.04–0.18);	[36]
**Nutrients**					
Dietary fiber	Cross-sectional study	459 hypertensive patients	<8.1 g/day vs. ≥15.4 g/day	Associated with a higher incidence of depression (OR, 2.757; 95% CI, 1.035–7.346);	[26]
n-3 fatty acids	Cross-sectional study	12,268 adults	The highest vs. the lowest quintile	Inversely associated with anxiety EPA: OR, 0.82; 95% CI, 0.69–0.98 DHA: OR, 0.83; 95% CI, 0.69–0.98 DPA: OR, 0.82; 95% CI, 0.69–0.98;	[107]
Branched-chain amino acids	Cross-sectional study	3175 Iranian adults 18 ≤ age ≤ 55	The highest vs. the lowest tertile	Associated with a lower risk of anxiety (OR, 0.66; 95% CI, 0.47–0.91);	[105]
Vitamin B_6_	Cross-sectional study	3362 adults	The lowest vs. the highest tertile	Associated with a higher risk of anxiety (OR, 2.30; 95% CI, 1.19–4.46);	[106]

CI, confidence interval; OR, odds ratio; HR, hazard ratios. EPA, eicosapentaenoic acid; DPA, docosapentaenoic acid; DHA, docosahexaenoic acid.
